# Human T-lymphotropic virus type 1(HTLV-1) integration, HTLV-1-associated infective dermatitis (IDH) and the risk of Adult T cell leukaemia/lymphoma (ATLL)

**DOI:** 10.1186/1742-4690-8-S1-A50

**Published:** 2011-06-06

**Authors:** Carol Hlela, Nicolas Gillet, Charles Bangham, Teresa Marafioti, Graham Ogg

**Affiliations:** 1 MRC Human Immunology Unit, University of Oxford, Weatherall Institute of Molecular Medicine, John Radcliffe Hospital, Oxford, UK; 2 Molecular and Cellular Epigenetics, University of Liège (ULg), Liège, Belgium; 3 Immunology Department, Imperial College, London, UK; 4 Pathology Department, University College London, London, UK

## 

HTLV-1 associated infective dermatitis (IDH) is a chronic dermatitis that affects a proportion of HTLV-1 infected children. IDH serves as an early clinical marker for HTLV-1 and may be associated with an increased risk of other HTLV-1 associated conditions such as adult T cell leukaemia/lymphoma (ATLL) and HTLV-1-associated myelopathy/transient spastic paraparesis (HAM/TSP). Since a high proviral load is found in all 3 diseases, we wished to characterise HTLV-1 replication in children with IDH. We studied a cohort of 10 cases with IDH and two asymptomatic carrier (AC) individuals from South Africa. We found high levels of the HTLV-1 provirus in the skin: mean value was 47.09 proviral copies per 1000 cells, compared with 137 proviral copies per 1000 cells in blood. Staining sections from IDH skin lesions revealed strong intracytoplasmic staining of HTLV-1 Gag protein but not Tax protein. To study the clonality of HTLV-1 replication in IDH, we used a recently developed high throughput Illumina sequencing protocol [1] to map and quantify the proviral integration sites in skin and blood of patients with IDH. Data from IDH patients were compared with data from another coinfection (Strongyloidiasis) and with an existing large data base of clonality in patients with HAM/TSP, patients with ATLL and ACs. The results suggest that preferential integration of the provirus in transcriptionally active regions of the genome, which has been demonstrated in ATLL, HAM/TSP and ACs, was lacking in IDH. We conclude that in IDH, additional selection forces promote oligoclonal expansion which override the normal pattern of selective T-cell expansion. We postulate that these selection forces are exerted by Staphylococcus aureus antigens or superantigens present in IDH. Long-term persistent stimulation of T-cell expansion may predispose to ATLL.
